# Understanding the Liver-Stage Biology of Malaria Parasites: Insights to Enable and Accelerate the Development of a Highly Efficacious Vaccine

**DOI:** 10.4269/ajtmh.17-0895

**Published:** 2018-08-20

**Authors:** Annie X. Mo, Glen McGugan

**Affiliations:** Division of Microbiology and Infectious Diseases, National Institute of Allergy and Infectious Diseases, National Institute of Health, Bethesda, Maryland

## Abstract

In August 2017, the National Institute of Allergy and Infectious Diseases convened a meeting, entitled “Understanding the Liver-Stage Biology of Malaria Parasites to Enable and Accelerate the Development of a Highly Efficacious Vaccine,” to discuss the needs and strategies to develop a highly efficacious, whole organism–based vaccine targeting the liver stage of malaria parasites. It was concluded that attenuated sporozoite platforms have proven to be promising approaches, and that late-arresting sporozoites could potentially offer greater vaccine performance than early-arresting sporozoites against malaria. New knowledge and emerging technologies have made the development of late-arresting sporozoites feasible. Highly integrated approaches involving liver-stage research, “omics” studies, and cutting-edge genetic editing technologies, combined with in vitro culture systems or unique animal models, are needed to accelerate the discovery of candidates for a late-arresting, genetically attenuated parasite vaccine.

## INTRODUCTION

Development of a highly efficacious malaria vaccine with at least 75% protective efficacy is one of the research and development (R&D) targets identified by both the Global Vaccine Action Plan^1^ and the Malaria Vaccine Technology Roadmap.^2^ The most advanced vaccine product to date, however, RTS,S/AS01, developed by GlaxoSmithKline, elicits only a moderate, short-lived level of protection against clinical malaria (33–50%) in older infants.^3^ Thus, the development of a second-generation malaria vaccine with improved efficacy is a crucial global health priority. In addition, vaccines that reduce transmission and target both *Plasmodium falciparum* (*Pf*) and *Plasmodium vivax* (*Pv*) will become increasingly important, as global malaria control and elimination efforts approach their end game.

The malaria liver stage represents a population bottleneck and, as such, is an attractive vaccine target for both *Pf* and *Pv*. In recent years, significant progress has been made in the development of whole sporozoite vaccine strategies targeting the liver stage of *Pf* parasites. These include radiation-attenuated sporozoites (RAS), genetically attenuated sporozoites (GAP), chloroquine prophylaxis with (live) sporozoites (CPS), and the use of murine parasite *Plasmodium berghei* (*Pb*) as a platform to deliver human *Pf* parasite antigens (Ags) (*Pb*/*Pf*).^4^ Evaluation of these strategies in a controlled human malaria infection (CHMI) setting indicated that CPS immunization using live sporozoites was much more effective at eliciting protection (> 75%) compared with RAS immunization; protective immunity elicited by CPS is believed to be directed against preerythrocytic (most likely liver-stage) parasites.^5–7^ This increased potency is thought to reflect the greater biomass and diverse Ag expression of live *Pf* parasites that can replicate in the liver for at least 6–7 days before being killed by drug treatment, in contrast to RAS which only develop in the liver for approximately 2–3 days.

Genetically attenuated sporozoites approaches offer opportunities to arrest sporozoites at certain times of their liver-stage development. There are ongoing efforts in multiple laboratories to develop and test several such approaches in *Pf*, especially GAPs that can support longer liver-stage development. To date, however, only mutations that arrest parasite development at the early-liver stage have been made, and the development of a stable *Pf* GAP with late liver-stage replication comparable with that of CPS has not been successful. Undoubtedly, there remain knowledge gaps in the liver-stage biology for both *Pf* and *Pv* in human infections as compared with what has been observed in rodent malaria infections in animal models. To address these, a workshop was convened to bring together investigators from different research areas, including sporozoite vaccine R&D, malaria immunology, malaria liver-stage biology, and molecular parasitology to discuss the current state of whole sporozoite vaccines, the liver-stage biology and immunology of malaria parasite infections, and to brainstorm about scientific and technical approaches to accelerate research in priority areas. The goal was to identify targets and strategies to assist in the design and development of a safe, efficacious GAP vaccine against malaria.

## STATUS OF SPOROZOITE VACCINE R&D

During the workshop, progress on the development of whole parasite/sporozoite-based vaccines targeting liver stages was summarized. Various forms of attenuated sporozoite vaccines delivered via direct venous inoculation (DVI) have been demonstrated to be safe, tolerable, and efficacious. Both RAS and CPS immunizations showed a significant level of short-term sterile protection against both homologous and heterologous parasite strains under CHMI in malaria-naïve adults in the United States. Protection remained significantly high against a homologous strain of parasite 1 year later but diminished significantly against a heterologous strain.^8–10^ In a natural exposure setting in Mali, where the parasite attack rate is more than 90%, vaccination conferred 29% protection by proportional analysis but 52% protection by time-to-event analysis.^11^ Of note, in a Phase IIb trial in Kenya where the attack rate was 49%, the RTS,S/AS01 vaccine achieved only 29% protection in a similar time-to-event analysis.^12^ Currently, the RAS vaccine is being tested in 5- to 12-month-old infants in Tanzania. Clinical trials to optimize the regimen and address other vaccine-related issues needed to bring the RAS product to licensure and deployment are underway or in the planning stage.

Quantitatively, although immunization with three doses of 5 × 10^4^ live sporozoites (at 4-week intervals) under chloroquine coverage (CPS) provided 100% protection for 10 weeks,^7^ three doses of 4.5 × 10^5^ sporozoites were needed for 87% protection with RAS immunization.^9^ Therefore, it appears that at least more than 9-fold more RAS sporozoites are needed to achieve similar levels of protection with live sporozoites immunization. Although live infectious sporozoites seem to be highly efficacious, they will not be an ideal vaccine candidate because of their pathologic potential and suboptimal manufacturing consistency. The GAP approach, on the other hand, introduces stably arrested sporozoites at defined liver-stage timepoints and presents an attractive alternative for sporozoite vaccine development. The greater efficacy of live sporozoites has been attributed to their prolonged liver-stage development, enabling them to replicate and form a larger biomass that expresses greater amounts of diverse protein Ags and, thus, induces potentially broader and longer lasting immune stimulation. In fact, in mouse models, a *Pb* late-arresting GAP (LAGAP) vaccine showed not only a much superior level of protection as compared with an early-arresting GAP (EAGAP) sporozoite vaccine, but also cross-species and cross-stage protection. The *Pb* LAGAP also expressed a much larger repertoire of parasite proteins and higher levels of protective CD8 T cells.^13^ Therefore, a *Pf* LAGAP resembling the *Pb* LAGAP would potentially represent an ideal vaccine candidate for malaria control and elimination.

Generation of a *Pf* LAGAP remains a great challenge, however. The biggest hurdle is to ensure safety, that is, no leakage of erythrocyte-infective merozoite progeny into the blood when parasite development is approaching the end of the liver stage. Two different *Pf* GAP sporozoites have been tested in humans via mosquito bite delivery, all of which arrested early in the liver stage (days 2–3).^14,15^ Unfortunately, due to attenuation “leakage,” the P53^-^P36^-^ double-knockout GAP produced parasitemia in one vaccinated subject. In a recent Phase I trial, however, the P53^-^p36^-^Sal1^-^ triple-knockout GAP produced no such “leakage.” Several laboratories are attempting to generate a LAGAP but thus far have not reported success. It was pointed out that basic research to investigate the mechanism of P53^-^/P36^-^ GAP attenuation leakage, or parasite liver-stage arrest or “breakthrough” in general, may help guide future GAP development.

The latest research progress on *Pv* whole sporozoite vaccines was also reviewed. Importantly, whole *Pv* sporozoite vaccine development and *Pv* liver-stage basic research have both been significantly hampered by the lack of a continuous, long-term, in vitro liver-stage culture system, and have relied on primary human hepatocytes or nonhuman primate (NHP) malaria liver models. *Plasmodium cynomolgi* exhibits relapse features in NHPs similar to that of *Pv* in humans, and has been used to study relapse biology. The extent of its relevance to *Pv* relapse biology remains unknown. As for *Pv* whole sporozoite vaccine development, the current strategies are limited to the following: 1). using specific pathogen–free NHP as a “factory” to grow live *Pv* sporozoites for radiation attenuation; 2). exploring *Pf* RAS for potential cross-species protection in clinics; or 3). using *Pb* or *Pf* sporozoites as a backbone to genetically introduce select *Pv* Ags. To facilitate *Pv* whole sporozoite vaccine development, the following *Pv* research priorities were strongly emphasized: establishing an in vitro culture system; discovering infectivity genes in the *Pv* genome; and understanding liver-stage hypnozoite biology and relapse control.

In summary, it was unanimously agreed that attenuated sporozoite platforms provide a promising approach for generating a highly efficacious vaccine for malaria, and an LAGAP, if made, could have the potential to provide a far superior performance over current RAS or GAP vaccine candidates. Although current attenuated sporozoite vaccine development efforts should not be slowed down, priority should be placed on using existing technologies and knowledge to produce a potentially highly efficacious LAGAP. Understanding the mechanisms of liver-stage “arrest” and “breakthrough” are important for designing future GAP mutants. Of note, it was pointed out that several definitions have been used to define preerythrocytic vaccine efficacy for different trials or different vaccine candidates, including sterile protection, delayed patency (use time-to-event analysis based on infection/parasitemia), or clinical malaria incidence. Great emphasis was placed on the need to harmonize the definition of vaccine efficacy to ensure accurate assessments and comparisons of vaccine performance.

## THE IMMUNOLOGY OF SPOROZOITE VACCINES

The immunological mechanism underlying sporozoite vaccine protection is much more complex than originally thought. Accumulated clinical data have demonstrated that protective immunity is a combined immune signature rather than a single immune parameter in a human setting. The specific immune profile of the recognized Ags could be quite heterogeneous among individuals; therefore, whole sporozoite vaccines have a much higher chance of success than subunit vaccines with a narrow Ag focus. It was shown that trained innate immunity could also reduce liver-stage parasite infection and development but only for a short duration. Antibody (Ab) and effectors of cellular immunity, including CD4, CD8, natural killer cells, γδ T cells, or other polyclonal T cells, are involved in *Pf* sporozoite vaccine protection. The level of CD8 T cells in blood following immunization, however, has been very low, perhaps due to inefficient or suboptimal assays to measure CD8 T cells ex vivo or in vitro. As such, the exact role of human CD8 T cells for vaccine protection has not been confirmed. A better assay to measure sporozoite-specific CD8 T cells is needed, and the relationship between CD8 T cells in the blood and CD8 T cells in the liver needs further exploration. In addition, the level of Ab response was consistently observed to be much lower in African adults as compared with malaria-naïve adults in the United States, and many CD8 T cells in individuals in endemic areas recognize blood-stage Ags unrelated to preerythrocytic immunity. Efforts should be directed at gaining a better understanding of the underlying immunological reasons for the decreased vaccine efficacy observed in endemic areas as compared with the United States.

Protective immunity in animal models, on the other hand, has been rather well characterized. Even though NHPs are not a natural host for *Pf*, data obtained from NHP studies appeared to be predictive of human vaccine efficacy. Direct venous innoculation of RAS, unlike intradermal (ID) or subcutaneous (SC) immunization, proved to be efficacious in humans and also induced significantly higher levels CD4 and CD8 T cells in the liver of NHPs as compared with ID or SC routes. In mice, besides Ab and CD4 T cells, CD8 T cells were consistently shown to be the most important protective immune factor, functioning as the most specific effectors through cognate hepatocyte recognition, Ag presentation, and secretion of interferon-γ, tumor necrosis factor, or perforin. An extremely high frequency of circulating CD8 T cells is required for sterile protection. Recently, rather than the conventional CD8 central memory T_CM_ or CD8 effector memory T_EM_ cells, CD8 tissue resident memory T_RM_ cells were observed to constantly patrol sinusoids, exert a strong effector function, and be essential for sterile protection.^16^ T_RM_ cells have been shown to form spontaneously on RAS immunization and can be enhanced by inflammation (e.g., adjuvant) or Ags in tissues. T_RM_ cells can also recruit T_CM_ and T_EM_ cells to the liver as a backup strategy. Most importantly, initial priming was thought to occur in the spleen and liver-draining lymph nodes but not in the liver on RAS immunization by DVI; however, the liver is still an important site because it may generate Ags for priming to occur. Subsequent boosting may convert primed cells into T_RM_ cells. Further knowledge about T_RM_ formation, function, durability, and maintenance would help improve whole sporozoite vaccine design and vaccination strategies. Nevertheless, it was noted that there are still gaps in translating findings from mouse models to human settings. Research on human T_RM_ cells in general is lacking and studies on the anatomic sites and cellular/molecular events for priming and boosting T_RM_ cells during sporozoite immunization would be very helpful in designing a better sporozoite vaccine and relevant vaccination strategy.

There were active discussions that LAGAP may generate some degree of blood-stage immunity which might skew host immune responses toward those that are more strain specific; no evidence of this was found in mouse models of infection, however. The consensus was that rather than being detrimental to preerythrocytic stage immunity, the blood-stage immunity elicited by LAGAP, if any, could be an added benefit. Understanding the link between liver-stage biology and liver-stage immunity, and the difference between EAGAP-specific and LAGAP-specific T cells, including their Ag specificity, phenotype, and functionality, were identified as key areas of investigation. It was also suggested that defining vaccine efficacy using time-to-event analysis could be meaningful in measuring immunological benefit because delayed infection/parasitemia could reflect more late liver stage Ag expression for immune priming or more time for specific immunity to develop and mature. In addition, the immunological mechanisms associated with differential vaccine efficacy between malaria-naïve adults in the United States compared with African adults with a history of prior malaria exposure should be further explored. Finally, developing assays to measure human CD8 T cell responses with better specificity and sensitivity, identifying CD8 T cell epitopes and protective Ags for Ab recognition, are needed not only to improve our understanding of sporozoite vaccine protection mechanism, but also to better select more informative immunological endpoints for clinical trial evaluation.

## LIVER-STAGE BIOLOGY AND “OMICS”

*Plasmodium* parasites have evolved a complex life cycle with the sporozoite constituting the most versatile and enigmatic form of the parasite, being the stage formed in the mosquito vector and then undergoing further differentiation in human liver cells. Following the bite of an infected mosquito, highly motile sporozoites leave the bite site, travel through the circulation until they find and invade hepatocytes. Once hepatocyte invasion is complete and infection is established, the complex process of parasite liver-stage development is initiated, transforming the parasite from an invasive extracellular form to an intracellular trophozoite within a parasitophorous vacuolar membrane (PVM), culminating in the production of invasive exoerythrocytic merozoites. Along the liver-stage journey, the parasite encounters many different cell types and conditions, coupled with commensurate changes in parasite gene expression and morphology. For *Pf*, this process takes 6–7 days. *Plasmodium vivax* sporozoites can also differentiate into a dormant form, the hypnozoite, which can remain latent for months to years before reactivating, developing further in the liver, and causing blood-stage infection, thus, accounting for the periodic relapses seen in vivax malaria.

Understanding the molecular and cellular events during *Pf* and *Pv* liver-stage development becomes extremely important in designing an attenuated sporozoite vaccine that arrests precisely at a desirable time point of liver-stage development. Since the discovery many years ago of the exoerythrocytic development of mammalian malaria parasites in the liver, however, much of our knowledge of this stage has been gained from studies performed with rodent malaria in animal models. The panel discussed the surprisingly limited knowledge of *Pf* and *Pv* liver-stage biology and noted that studies on molecular mechanisms surrounding merozoite invasion, development, and egress in red blood cells (erythrocytic phase) have been far ahead of those investigating sporozoite invasion of hepatocytes, intrahepatic development, and subsequent egress. This is in part because liver infections are asymptomatic and cannot be readily observed during natural human infections. In addition, *Pf* and *Pv* have a narrow host preference (i.e., are restricted to hepatocytes of humans and great apes), and a natural sporozoite infection of human hepatocytes usually results in the invasion of only a small number of hepatocytes. It is anticipated that the recent development of in vitro human hepatocyte platforms and liver-humanized mice would greatly aid in liver-stage *Pf* and *Pv* basic research.

Some rodent parasite proteins involved in PVM formation were described to be essential for liver-stage formation and development, including proteins that transport lipids and iron across the PVM to support parasite growth. A more comprehensive understanding of the identity and function of all such proteins is still needed. Inside hepatocytes, there is a massive increase in parasite biomass, suggesting active anabolic pathways in addition to nutrients acquired from the metabolically active host cell. One of these pathways has been demonstrated to involve de novo fatty acid synthesis, although the exact role of these fatty acids remains largely unknown.

Creating LAGAPs for clinical use as a vaccine requires the identification of candidate genes that, when knocked out, will completely block late-stage parasite development. Several “omics-based approaches” have previously been used to identify liver-stage targets, including cDNA sequencing, RNA sequencing (including dual RNAseq for both parasite and host), proteomics and other bioinformatics approaches, and high-throughput (HTP) reverse genetic screens. Again, such studies have largely been performed in rodent malaria parasites and are not completely predictive of human malaria parasites. Thus, there remains an extensive knowledge gap regarding *Pf* and *Pv* liver-stage parasite differentiation, gene transcription, protein expression, and functional identification. At the cellular level, knowledge of *Pf* and *Pv* parasites’ interplay with host hepatocytes is also limited. Better transcriptomic and proteomic data for both *Pf* and *Pv* are needed. Given the current availability of human hepatocyte lines in 2D or 3D culture systems, primary human hepatocyte cultures, and severe combined immunodeficiency mice engrafted with human hepatocytes, identification of genes controlling parasite arrest in human liver should be possible. Suggested approaches included studying *Pf* gene transcription or expression profiles across life cycle stages to identify those that are highly expressed in the liver stage but less or not at all in other life cycle stages and detailed time course studies of parasite gene expression in infected hepatocytes to dissect gene expression regulation. The panel noted, however, that the most abundant proteins are not always the most important or the best targets. The egress pathway was also suggested as a priority target because drugs targeting egress allow parasites to leave the vacuole but remain in the host cell, and the egress process in liver-stage parasites seems distinct from that in blood-stage parasites. Exploring compounds that specifically interfere with hepatic stage development to extract information for genetic arrest was also proposed. Another suggestion was to study “master regulatory genes” and consider HTP deletion methods to identify late-arresting phenotypes, or use comparative biology approaches to identify similar/conserved genes/pathways that control parasite proliferation, differentiation, or egress.

Several immediate tasks were repeatedly emphasized by the panel. Because the production of vialed, live *Pf* sporozoites is now technically feasible, these should be made readily available to the research community to enable and facilitate worldwide basic research, especially on the *Pf* liver stage. In addition, although green fluorescent protein-labeled *Pf* is routinely used for microscopy, it is highly problematic for the sorting of infected hepatocytes for downstream omics studies because of a high signal to noise ratio. As such, transgenic parasites expressing a lower noise fluorescent protein for such studies would be a valuable resource for the research community. Further, systematically obtaining *Pf* liver-stage transcriptomic information, facilitated by substantially reduced costs of transcriptomic analysis and availability of single-cell transcriptomic analyses, would greatly enable liver-stage research. Finally, research on *Pf* liver-stage biology has been hampered in part by low hepatocyte infection rates and incomplete development in current in vitro models. Although parasite development is more complete using a humanized mouse model, high costs and technical challenges remain issues. As such, an improved, more easily accessible system, supporting the growth and full development of the liver-stage parasite is needed.

In summary, a better understanding of liver-stage biology and sporozoite gene function is needed for targeted gene deletion strategies. There is a current need to create better liver stage transcriptomes and proteomes for *Pf* and *Pv*. Empirical approaches for identifying targeted arrest genes may consider the egress pathway, “master regulatory genes,” chemically validated lethal genes, or other genes and pathways identified using various model organisms.

## GENETIC MANIPULATION OF MODEL ORGANISMS

Given the limited availability of *Pf* liver-stage–“omics-based studies” and the less tractable features of human malaria parasites, the use of functional genetic screens in various model organisms to gain coordinated insight into the identity of appropriate target genes would be useful in predicting and down-selecting targets for further investigation. Although rodent malaria models, and the ready availability of reagents and screening tools associated with them, provide the most convenient approach to gain useful biological information, limitations in translating findings in rodents to humans were noted. For example, development of rodent *Plasmodium yoelii* sporozoites with gene deletions in fatty acid biosynthesis pathway was arrested late in the liver stage, whereas gene ortholog deletions in *Pf* turned out to be lethal for the parasite. The use of model organisms, such as the more genetically tractable apicomplexan, *Toxoplasma*, could also be informative for several reasons. Chiefly, there appears to be a high correlation between expression profiles of essential genes in *Toxoplasma* and those of human malaria parasites,^17^ suggesting the potential predictive value of *Toxoplasma* transcriptomics. In addition, *Toxoplasma* is more genetically tractable than *Plasmodium*, in part, due to its higher transformation efficiency and higher germinal center content, which allows for pooled genetic screens and genome-wide phenotypic analyses following reverse genetic engineering. In fact, many dispensable and essential genes of *Toxoplasma* have been identified, and the corresponding complex phenotypes have been further dissected using conditional knockout strategies and conditional functional screens. Another apicomplexan genus, *Cryptosporidium*, is considerably less tractable than *Toxoplasma* and has been difficult to culture in vitro, hampering genetic manipulation. Despite this limited tractability, advances have been made in genetic manipulation and in understanding the biology of this parasite. Although not as directly predictive as studies in *Toxoplasma*, lessons learned from *Cryptosporidium* may inform efforts for *Pf* and *Pv* culture and manipulation. Comparative biology research using knowledge gained from the study of these model organisms would likely yield useful information that may guide relevant research efforts in human malaria parasites.

Various methodologies have been used in reverse genetic approaches, including zinc finger nucleases, transcription activator-like effector nucleases, and clustered regularly interspaced short palindromic repeats (CRISPR)/Cas9. Recently, CRISPR/Cas9 editing has been used successfully in *Pf* to create deletion mutant parasites and is expected to be a valuable tool in LAGAP sporozoite generation, due to increased efficiency and HTP potential, as compared with homologous recombination alone. It is generally thought that repair of double-stranded breaks in *Pf* only occurs through homologous recombination with the addition of a repair template, due to the lack of nonhomologous end-joining. The requirement for this template further decreases the likelihood of off-target effects in *Pf*. Regardless of the mechanism used, more efficient transfection and gene deletion/alteration strategies, and better molecular tools and cellular systems, would significantly improve the HTP efficiency of the genetic manipulation of *Plasmodium* parasites.

It was highlighted that the technical process chosen for generation of mutant parasites must consider safety and regulatory requirements for future downstream vaccine development. Certainly, the clean deletions of candidate genes that are selection marker-free is an important consideration. Additional features to enhance safety include kill switches or built-in auxotrophy for biocontainment. Furthermore, the selection of *Pf* strains other than NF54 is an important topic and should be carefully considered. A major advantage of NF54 sporozoites is that manufacturing and production data for future development are readily available, yet it is worth considering whether other *Pf* strains may offer better genetic manipulation characteristics and potentially better vaccine candidacy. The panel also emphasized that one gene deletion may be insufficient and two or more gene disruptions (including combining early-, middle-, and late-arresting genes) may be required to produce the most effective product. Finally, it was highly recommended that once generated, all mutant cell lines be systematically cataloged, deposited into a repository, and preserved for the research community at large, similar to the malaria box for drug development. This should also include down-selected or unpublished mutants, such as those not leading to protection, or those producing an undesirable phenotype or no discernible phenotypic changes.

## TECHNOLOGIES FOR GAP SELECTION

To ensure sufficient parasite biomass for efficient priming of host immunity, an LAGAP should ideally arrest in the liver at days 4–5 or beyond. Initial assessment of liver-stage genetic mutants should include the abilities to complete blood- and mosquito-stage development, productively infect hepatocytes, and produce gametocytes to ensure production of sporozoites. Once confirmed, the most critical features of these mutants are safety and potency, which can be evaluated using humanized mouse models or microculture systems.

For rodent sporozoites, mouse models provide the most convenient approach to evaluate breakthrough and protection efficacy of LAGAP. However, it was highlighted that data need to be interpreted carefully because mouse models and parasites are intrinsically variable and can show various levels of attenuation depending on the mouse or parasite strains, which could easily create misleading data and safety concerns. For human GAP mutants, HTP approaches using culture systems to down-select genetic mutants is now considered possible. Long-term primary human and NHP hepatocyte cultures have been successfully established. In these cultures, which were originally developed for *Pv* liver-stage anti-hypnozoite drug screening, *Pf* sporozoite infectivity has been high enough (5%) for an initial HTP effort of LAGAP screening.^18^ Apparently, further optimization and refinement are still needed. Other in vitro culture systems that need improvement include the hepatocyte cell line HC-04, which thus far has only supported early sporozoite growth, and hepatocyte culture, which allows only suboptimal sporozoite infectivity. Regardless, tissue culture screening can only be used as a HTP approach for initial down-selection of candidate mutants. Breakthroughs observed with cultured cell lines may not resemble the in vivo situation. Further confirmatory studies need to be carried out, for example, with a humanized mouse model to confirm lack of breakthrough before clinical trials. Some potential cost-saving approaches, such as multiplex infection using genotype barcoding, were suggested. In addition, the cost of the humanized mouse model may soon be significantly reduced by improved technology to generate copious quantities of human primary hepatocytes for mouse engraftment. As such, direct testing in the humanized mouse model at a relatively large scale without any initial tissue culture screening may soon be possible.

Regardless of assays or models used for candidate screening or validation, several experimental parameters need to be further fine-tuned or even standardized; for example, the quantification of liver-stage development using quantitative polymerase chain reaction and in vivo imaging, the definition of late liver-stage arrest (the death/arrest of sporozoites, the death or hypoxia of sporozoite-infected hepatocytes), and, as culture systems allow, the evaluation of merosome formation or merozoite emergence (breakthrough). Finally, the potency of selected mutants could be evaluated by immunogenicity in relevant animal models or Phase I clinical trials. In conclusion, critical assays or models are available, although further optimization is needed to fully characterize LAGAPs and ensure absence of breakthrough events before testing them in human clinical trials.

## CONCLUSION

Given the large genome of malaria parasites, sporozoites possess a large Ag repertoire which changes and increases as liver-stage development progresses and the corresponding parasite biomass increases. As such, an LAGAP can potentially induce a much higher level and better quality of protective immunity leading to better vaccine efficacy as compared with the current EAGAP. To reach this goal, a series of key challenges and opportunities (Table 1) were identified. With more targeted basic biology studies, detailed genomic analyses of liver-stage parasites, comparative genomic research with model organisms, advances in transcriptomic technologies and bioinformatics tools, and new HTP microculture systems and humanized mouse models, the possibility of designing, constructing, and testing desirable LAGAP becomes more feasible. A multidisciplinary approach to address the identified challenges (Table 1) through integrated R&D activities will greatly facilitate and accelerate the discovery of a highly efficacious malaria vaccine for the goal of malaria control, elimination and even eventual eradication.

**Table 1:** Key challenges and opportunities

Category	Key knowledge gaps, challenges, and opportunities
Sporozoite vaccines	Better understanding and harmonization of vaccine efficacy definition (sterile protection, time-to-event delay, and clinical malaria incidence) and how they reflect mortality and morbidity
Immunology	Assay development to measure human CD8 T cell responses
	Understanding the predictive value of blood CD8 T cells for liver-stage immunity
	Understanding human liver resident memory CD8 T cells
	Identification of epitopes for protective CD8 T cell and antibody responses
	Understanding the immunological basis (e.g., caused by coinfections) for differential vaccine efficacy between U.S.-naïve and African populations
Biology	Greater understanding of parasite liver-stage biology and development
	Interplay between parasites and hepatocytes
	Improved access to *Plasmodium falciparum* sporozoites
	Better human liver model systems
	How liver-stage biology is linked to liver-stage immunity
	Basic research on how “breakthrough” occurs
	For *Plasmodium vivax* (*Pv*):
	In vitro culture system
	Identification of *Pv* infectivity genes
	Understanding liver-stage hypnozoite biology and relapse control
“Omics” and model organisms	Transcriptomic analyses across the malaria parasite life cycle
	Comparative “omics” with model organisms
	More efficient transfection and gene deletion/alteration strategies
	Better molecular tools or cellular systems to enhance genetic manipulation efficiency
	Tools for multiplex genetic manipulation and genotype barcoding
Technologies for selection	Optimization and improvement of currently available primary human hepatocyte microculture and humanized mouse models
	Assessment of approaches to quantify liver-stage development (qPCR, in vivo imaging, etc.)
	Understanding “late-arresting” definition (death of parasite, death or hypoxia of infected hepatocytes, etc.)
	Establishment of multiplex screening systems
